# Structural neuroimaging correlates of social deficits are similar in autism spectrum disorder and attention-deficit/hyperactivity disorder: analysis from the POND Network

**DOI:** 10.1038/s41398-019-0382-0

**Published:** 2019-02-04

**Authors:** Danielle A. Baribeau, Annie Dupuis, Tara A. Paton, Christopher Hammill, Stephen W. Scherer, Russell J. Schachar, Paul D. Arnold, Peter Szatmari, Rob Nicolson, Stelios Georgiades, Jennifer Crosbie, Jessica Brian, Alana Iaboni, Azadeh Kushki, Jason P. Lerch, Evdokia Anagnostou

**Affiliations:** 1 Department of Psychiatry, University of Toronto, Toronto, ON Canada; 2 Clinical Research Services, The Hospital for Sick Children, and the Dalla Lana School of Public Health, University of Toronto, Toronto, ON Canada; 3 The Centre for Applied Genomics, The Hospital for Sick Children, Toronto, ON Canada; 4 Mouse Imaging Centre, The Hospital for Sick Children, Toronto, ON Canada; 5 The McLaughlin Centre and the Department of Molecular Genetics, University of Toronto, Toronto, ON Canada; 6 Department of Psychiatry, Neurosciences and Mental Health, The Hospital for Sick Children, Toronto, ON Canada; 7 Mathison Centre for Mental Health Research & Education, Hotchkiss Brain Institute, Cumming School of Medicine and Department of Psychiatry and Medical Genetics, University of Calgary, Calgary, AL Canada; 8 The Centre for Addiction and Mental Health, Toronto, ON Canada; 9 Department of Psychiatry, Western University and Children’s Health Research Institute, London, ON Canada; 10 Department of Psychiatry and Behavioural Neurosciences, McMaster University, Hamilton, ON Canada; 11 Department of Paediatrics, University of Toronto, Toronto, ON Canada; 12 Autism Research Centre, Bloorview Research Institute, Holland Bloorview Kids Rehabilitation Hospital, Toronto, ON Canada; 13 University of Toronto, Institute of Biomaterial and Biomedical Engineering, Toronto, ON Canada; 14 Department of Medical Biophysics, University of Toronto, Toronto, ON Canada

## Abstract

Autism spectrum disorder (ASD), attention-deficit/hyperactivity disorder (ADHD), and obsessive-compulsive disorder (OCD) have been associated with difficulties recognizing and responding to social cues. Neuroimaging studies have begun to map the social brain; however, the specific neural substrates contributing to social deficits in neurodevelopmental disorders remain unclear. Three hundred and twelve children underwent structural magnetic resonance imaging of the brain (controls = 32, OCD = 44, ADHD = 77, ASD = 159; mean age = 11). Their social deficits were quantified on the Social Communication Questionnaire (SCQ) and the Reading the Mind in the Eyes Test (RMET). Multivariable regression models were used to examine the structural neuroimaging correlates of social deficits, with both a region of interest and a whole-brain vertex-wise approach. For the region of interest analysis, social brain regions were grouped into three networks: (1) lateral mentalization (e.g., temporal–parietal junction), (2) frontal cognitive (e.g., orbitofrontal cortex), and (3) subcortical affective (e.g., limbic system) regions. Overall, social communication deficits on the SCQ were associated with thinner cortices in the left lateral regions and the right insula, and decreased volume in the ventral striatum, across diagnostic groups (*p* = 0.006 to <0.0001). Smaller subcortical volumes were associated with more severe social deficits on the SCQ in ASD and ADHD, and less severe deficits in OCD. On the RMET, larger amygdala/hippocampal volumes were associated with fewer deficits across groups. Overall, patterns of associations were similar in ASD and ADHD, supporting a common underlying biology and the blurring of the diagnostic boundaries between these disorders.

## Introduction

Social deficits are a defining feature of autism spectrum disorder (ASD), but also frequently affect children with attention-deficit/ hyperactivity disorder (ADHD)^1^, and can occur in obsessive-compulsive disorder (OCD) as well^2–5^. Increasingly, recognition of the overlapping and related nature of both the symptoms and the biology of different neurodevelopmental disorders^6–8^ has led to a call for research that spans diagnostic boundaries, and focuses instead on dimensions of psychopathology^9^.

Decades of neuroimaging research have begun to delineate the neural substrates of sociality. Different theoretical models of the social brain have been put forward from meta-analyses^10,11^ and reviews of the literature^12–14^ (summarized in Supplementary Table 1). Across models, three functional/structural groups emerge. Brain regions hypothesized to be involved in mentalization and empathy (group 1), cluster along the midline and lateral aspects of the brain, including the temporal–parietal junction (TPJ), superior temporal gyrus/sulcus (STS/STG), dorsal medial prefrontal cortex, temporal poles, and the posterior cingulate. Anterior and prefrontal regions (group 2), including the anterior and dorsal cingulate, orbital frontal cortex, and the dorsal and ventral lateral prefrontal cortices, may contribute to executive function and cognitive control over affective and social processes. Deeper cortical and subcortical structures (group 3), including the insula, amygdala, hippocampus, and the dorsal and ventral striatum, are more central to affective responding, memory, and social reward processing (Supplementary Table 1).

Structural neuroanatomical differences in many of these regions have been detected in individuals with OCD, ASD, and ADHD compared to controls^8,15–21^. For example, recent meta-analyses suggest increased frontal lobe thickness in ASD^20^, thinner temporal/parietal thickness in ASD and OCD^20,22^, and smaller subcortical volumes in ASD and ADHD^20,23^. The extent to which social deficits may differentially localize to specific brain regions/networks in different disorders is unclear, however^24^. In ASD, social deficits have been associated with both larger^25–29^ and smaller^27,30,31^ cortical measurements, particularly in frontal and temporal–parietal regions. In ADHD, social deficits were associated with greater overall cortical gray matter volume^32^ and smaller left caudate volumes^33^.

Cortical gray matter volume is a product of cortical thickness and surface area. Recent work has provided evidence that cortical thickness and cortical surface area measurements are under distinct genetic influences^34^, and follow unique developmental timelines^35,36^, necessitating that they be studied independently. Cortical thickness measurements on magnetic resonance imaging (MRI) are thought to reflect the underlying cortical microstructure, involving the number and organization of cortical neurons, neuronal dendritic arborization, the number and size of glial cells, and to some extent the maturation of the adjacent white matter^37^. Cortical thickness, including the timing and rate of cortical thinning, has been a major area of study across neurodevelopmental disorders, particularly in ASD^24^.

The association between brain (i.e., cortical thickness/subcortical volume) and behavior (i.e., social deficits) was the focus of the following study, in efforts to identify and compare the neural substrates of sociality across disorders. Given that the specific behavioral dimensions that contribute social impairments may vary across neurodevelopmental disorders (e.g., impaired mentalization in ASD^38^, executive function in ADHD^39^, and reward processing in OCD^40^), one hypothesis is that the neutral substrates of sociality will also differ by diagnosis (e.g., primarily lateral mentalization regions in ASD, frontal cognitive regions in ADHD, and subcortical regions OCD). An alternative hypothesis is that the brain regions associated with social deficits will span diagnostic boundaries. In children with ASD, ADHD, and OCD, for example, white matter fractional anisotropy correlated with adaptive functioning abilities, irrespective of diagnosis^8^. Neuroimaging analyses comparing the structural neuroanatomical correlates of social deficits across children with ASD, ADHD, or OCD have not yet been performed.

To address this knowledge gap, we first compared cortical thickness/subcortical volume measurements in social brain regions across a group of children with ASD, ADHD, OCD, or controls. Next, we examined how cortical thickness/subcortical volume corresponded with social deficits across disorders. We hypothesized that social deficits would correlate with structural anatomy, irrespective of diagnosis.

## Methods

### Participants

Participants were recruited via the Province of Ontario Neurodevelopmental Disorders Network, across four Centers in Ontario, Canada (Holland Bloorview Kids Rehabilitation Hospital, Toronto; The Hospital for Sick Children, Toronto; McMaster Children’s Hospital, Hamilton; and Lawson Health Research Institute, London). Controls were recruited through advertising in public transit, in hospitals, and on social media. Inclusion criteria were age <18 years, and a clinical diagnosis of ADHD, ASD, or OCD. Controls had no developmental diagnosis, and no first-degree family history of such. Participants were recruited into the study based on their primary psychiatric diagnosis; potential comorbid symptoms/traits were captured on symptom surveys (described below). Standardized research assessments confirmed the primary clinical diagnosis using established metrics (the Autism Diagnostic Observation Schedule—2^41^, the Autism Diagnostic Interview-Revised (ADI-R)^42^, the Kiddie-Schedule for Affective Disorders and Schizophrenia^43^, the Parent Interview for Child Symptoms^44^, and the Children’s Yale-Brown Obsessive-Compulsive Scale^45^). Research ethics board approval was obtained at each institution.

### Social metrics: The Social Communication Questionnaire and the Reading the Mind in the Eyes Test

The Social Communication Questionnaire (SCQ) is an established 39-item measure that quantifies risk for ASD through parent/caregiver report on social abilities and behaviors^46^. For our analyses, we focused on the 28 items assessing current and past social communication/interaction skills, excluding items on repetitive behaviors. The Reading the Mind in the Eyes Test (RMET) is a validated social perception task, in which study participants are asked to label the emotion/mental state in a still image of human eyes^47^. This metric quantifies social perception abilities. Participants in this study completed the child version of the RMET, which included at total of 28 items^48^. For simplicity and congruence with the SCQ, we examined the number of incorrect RMET items. Therefore, for both measures, higher scores indicate greater social deficits.

### Other measures

To characterize the study sample, ADHD symptoms were quantified across groups using the Child Behavior Checklist (CBCL) ADHD subscale. This is a parent report measure that yields age- and sex-normed *T*-scores. *T*-scores >65 are suggestive of elevated symptomatology^49^. To quantify OCD symptoms, the Toronto Obsessive-Compulsive scale was used. This is a parent report measure, where items are scored −3 to +3; total scores >0 discriminated OCD cases from controls in a community sample^50^.

### MRI and image analysis

All structural MRI data were collected at the Hospital for Sick Children in Toronto, between June 2012 and July 2017. The majority of scans (74%) took place on the 3-Tesla Siemens Trio TIM; a hardware upgrade to the Siemens Prisma scanner took place in June of 2015 (this affected 26% of the ADHD sample, 24% of the ASD sample, 62% of controls, and 7% of the OCD sample).

Cortical thickness measures were extracted from T1-weighted images using the CIVET pipeline (version 2.1.0)^51^ The CIVET pipeline applies a non-uniformity correction on the images^52^ followed by stereotaxic registration to the Montreal Neurologic Institute (MNI ICBM152) template (non-linear sixth-generation target)^53,54^. Next, brains were masked, extracted, and classified into gray matter, white matter, and cerebrospinal fluid, which were used to generate gray and white matter surfaces^55–59^. A surface-diffusion kernel was applied^60^, and regions were registered to the automated anatomical labeling atlas^61–63^. Cortical thickness measurements were taken from the distance between the two smoothed surfaces^64,65^. Quality assurance was assessed at the time of the scan for motion artifact, and was analyzed through the CIVET quality control (QC) analysis pipeline. Scans that were flagged on the QC analysis were manually reviewed for quality, and potentially excluded. Thickness measurements from the automated anatomical labeling atlas regions were grouped into regions of interest to reflect the three primary social neural networks^10,12–14^ (see Supplementary Table 1). We computed the vertex-wise thickness for each combined region of interest, and also examined the individual vertices across the whole brain. Subcortical structures were segmented using multiple automatically generated templates (MAGeT) and their volumes determined from the segmentation results^66^. For the region of interest analysis, we examined the association between mean thickness/volume measurements and social deficits in both the left and right hemispheres combined, except where previous data suggested significant lateralization effects (i.e., the temporal–parietal region^24,29,67^, the amygdala^68,69^, the dorsal striatum^33^, and the insula^70^].

### Statistical analyses

Statistical analyses were performed using SAS (University Edition) version 9.4, and R version 3.5.1.

As a preliminary analysis, we initially tested for diagnostic differences in brain structure for the combined social regions of interest, as well as mean whole-brain cortical thickness and volume. For this, we used a linear regression model to estimate the least-squares (LS) mean cortical thickness (in mm) by region, or mean volume (mm^3^) for subcortical structures, treating diagnosis (ASD, ADHD, OCD, or control) as a categorical predictor variable, while also including age, sex, and hardware upgrade as covariates to account for their effects.

Next, we used a logistic regression model to examine the proportion of items selected on the social measures (SCQ score/28 items, or RMET incorrect score/28 items) as the dependent variable, predicted by each millimeter increase in cortical thickness, or 0.1 cm^3^ increase in subcortical volume. Regions were examined in the three structural/functional groups. Odds ratios (ORs) are presented; ORs >1 indicate greater deficits with increasing size, whereas ORs <1 indicate fewer social deficits with increasing size. We included age, sex, hardware upgrade, and diagnosis as covariates, while also testing for interactions between thickness/volume and diagnosis. Where the thickness/volume-by-diagnosis interaction term was non-significant (*p* > 0.05), the interaction term was dropped from the model and ORs were presented across all groups combined. Where the interaction term was significant, ORs were presented for the diagnostic groups separately. Logistic regression analysis was chosen given that scores on the SCQ and RMET have a ceiling/floor, and follow a binomial distribution. Results are presented without correction for multiple comparisons in order to convey overall patterns; associations that remained significant after a false discovery rate (FDR) correction^71^ are indicated in the text/tables.

As a secondary analysis, the association between cortical thickness and social behavior was examined using a whole-brain vertex-wise approach, with the same logistic regression models applied to 70,000 vertices across the entire brain. ORs for vertices that remained significant after an FDR correction (*q* < 0.05) are presented graphically. The proportion of significant voxels, and the number of significant peaks (i.e., significant statistical maxima within a five vertex radius) per region of interest are also presented.

## Results

### Demographic characteristics

Of 343 initial scans, 31 (9%) were excluded due to low quality. Demographic data for all remaining participants are presented in Table 1. As expected, the ASD group had the highest level of social deficits on the SCQ and the RMET. All groups separated in terms of ADHD symptoms on the CBCL (ADHD > ASD > OCD > controls). The ADHD and control groups were similar in terms of OCD symptom severities. Correlations between behavioral measures are presented in Supplementary Table 2.

**Table 1:** Participant Demographics

	Overall	ASD (*n* = 159)	ADHD (*n* = 77)	OCD (*n* = 44)	Control (*n* = 32)	p-value^a^	Pairwise
**Females** n (%)	71 (22.8)	30 (18.9)	9 (11.7)	15 (34.1)	17 (53.1)	<0.0001	
**Age** Median (IQR) [min, max]	11.6 (9.4, 14.1) [6.2, 18.0]	11.7 (9.2, 14.4) [6.2, 18.0]	10.4 (9.0, 13.2) [6.5, 17.0]	12.5 (10.8, 14.9) [8.1, 17.7]	10.9 (8.4, 13.5) [6.1, 17.6]	0.01	ADHD vs. OCD p= 0.006 Other comparisons NS
**IQ** Median (IQR) [N]	100.0 (85.0, 111.0) [249]	93 (77, 107) [150]	104.5 (92.5, 113.5) [52]	118.5 (96.0, 127.0) [18]	110.0 (106.0, 119.0) [29]	<0.0001	ADHD vs. ASD p=0.003 ADHD vs. control: NS ADHD vs. OCD: NS ASD vs. control p<0.0001 ASD vs. OCD p=0.0004 Control vs. OCD: NS
**SCQ** Median (IQR) [N]	12.0 (5.0, 21.0) [294]	20.0 (15.0, 25.0) [150]	6.0 (3.0, 11.0) [73]	4.0 (2.0, 7.0) [40]	2.0 (1.0, 3.0) [31]	<0.0001	ADHD vs. ASD: <0.0001 ADHD vs. control: <0.0001 ADHD vs. OCD: NS ASD vs. control: <0.0001 ASD vs. OCD: <0.0001 Control vs OCD: 0.008
**SCQ Soc Com** Median (IQR)	8.0 (3.0, 14.0)	13.5 (9.0, 17.0)	4.0 (2.0, 8.0)	2.0 (1.0, 4.0)	2.0 (0.0, 3.0)	<0.0001	ADHD vs. ASD: <0.0001 ADHD vs. control: 0.0005 ADHD vs. OCD: 0.05 ASD vs. control: <0.0001 ASD vs. OCD: <0.0001 Control vs OCD: NS
**RMET** Median (IQR) [N]	18.0 (14.0, 20.0) (241)	16.0 (12.0, 19.0) [120]	18.0 (15.0, 19.0) [71]	21.0 (20.0, 22.0) [25]	19.0 (18.0, 20.0) [25]	<0.0001	ADHD vs. ASD: 0.05 ADHD vs. control: NS ADHD vs. OCD: 0.001 ASD vs. control: 0.004 ASD vs. OCD: <0.0001 Control vs. OCD: 0.004
**CBCL-ADHD** Median (IQR) [N]	63.0 (56.0, 72.0) [284]	65.0 (57.0, 72.0) [145]	69.0 (62.0, 74.5) [72]	57.0 (51.0, 67.0) [39]	50.0 (50.0, 51.0) [28.0]	<0.0001	ADHD vs. ASD: 0.003 ADHD vs. control: <0.0001 ADHD vs. OCD: <0.0001 ASD vs. control: <0.0001 ASD vs. OCD: 0.003 Control vs. OCD: <0.0001
**TOCS** Median (IQR) [N]	−3.0 (−30.0, 8.0) [264]	−2.0 (−21.0, 7.0) [143]	−20.0 (−45.0, −5.0) [73]	20.0 (8.0, 28.0) [41.0]	−41.0 (−60.0, −2.0) [7.0]	<0.0001	ADHD vs. ASD: <0.0001 ADHD vs. control: NS ADHD vs. OCD: <0.0001 ASD vs. control: 0.05 ASD vs. OCD: <0.0001 Control vs. OCD: <0.0001

SD: standard deviation. ASD: autism spectrum disorder, ADHD: attention-deficit/ hyperactivity disorder, OCD: obsessive-compulsive disorder. SCQ: Social Communication Questionnaire (n = 39 items). SCQ Soc Com: Social Communication Questionnaire Social and Communication items only (n = 28 items). IQ: Intelligence Quotient. RMET: Reading the Mind in the Eyes Task number of correct items out of 28. CBCL-ADHD: Child Behavior Checklist ages 6–18 ADHD Subscale T-score. TOCS: Toronto Obsessive-Compulsive Scale. Some participants had missing data for various measures; the number of participants with data available is presented in square brackets

^a^P-values are from the Kruskal-Wallis test (for continuous non-normally distributed data), or the chi-squared test (for categorical variables)

### No diagnostic differences in social brain structures between groups

The multivariable predicted LS means for the regions of interest for each diagnostic group are presented in Supplementary Table 3. There were no significant differences in brain structure between diagnostic groups with the exception of the hippocampus, where diagnosis had a nominal effect on hippocampal volumes (*F* = 4.10, *p* = 0.007, non-significant after FDR correction). On pairwise comparisons, the OCD group had larger mean hippocampal volumes. Also adjusting for whole-brain volume yielded a less significant main effect of diagnosis on hippocampal volume (*F* = 2.62, *p* = 0.05); other structures remained non-significant.

### Structural neuroimaging correlates of social communication deficits are similar in ASD and ADHD; OCD has some unique features

**Fig. 1:**
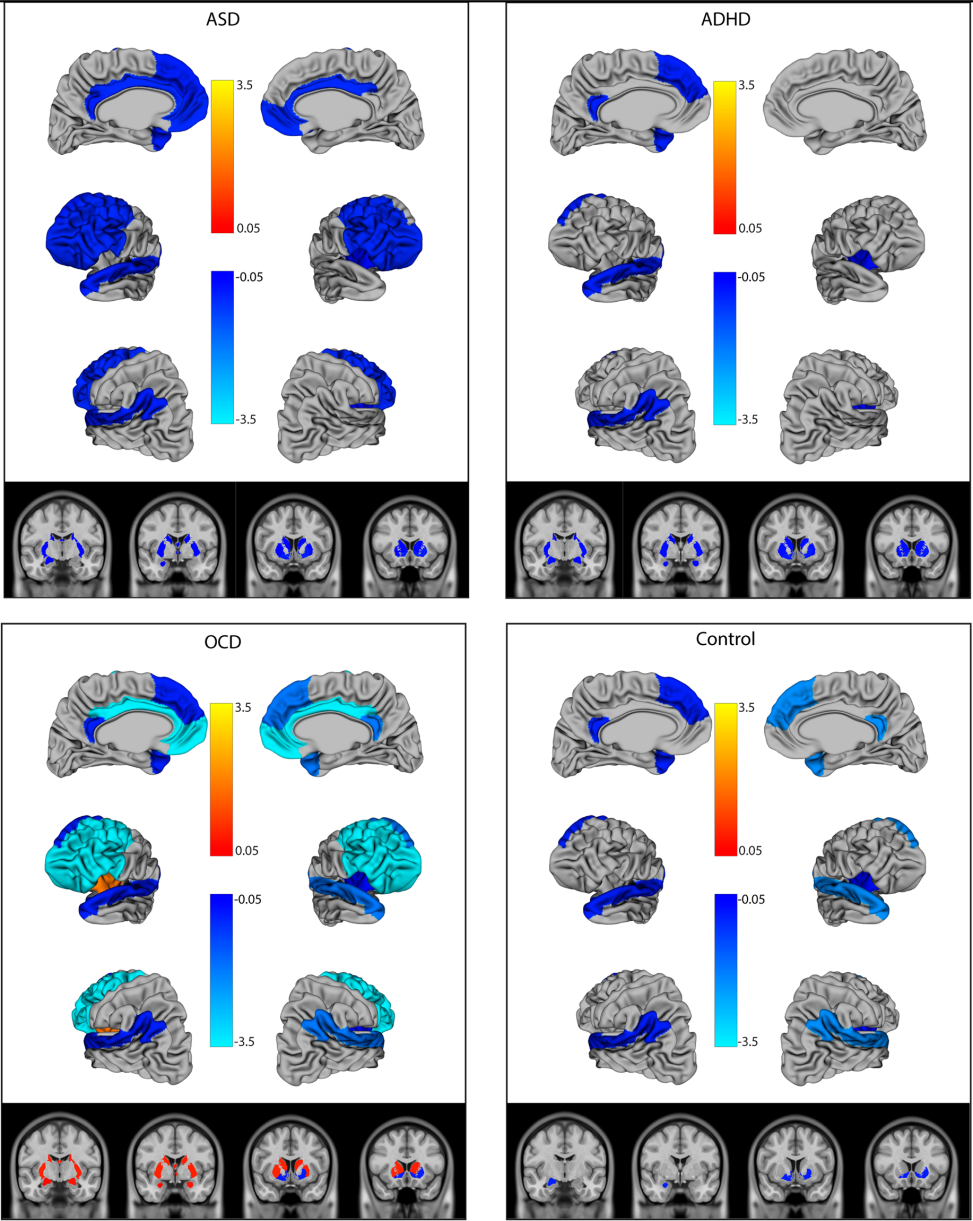
Log odds ratios of the association between brain structure in social regions of interest and social communication deficits on the SCQ. The figure shows the log odds ratios (ORs) for regions where *p* < 0.05 in the multivariable model. ORs indicate the change in SCQ score per increase in size (per mm increase in thickness, 0.1 cm^3^ increase in volume for the amygdala/hippocampus, or 1.0 cm^3^ increase in volume for other subcortical structure). Blue indicates ORs <1, where thicker/larger structures are associated with fewer deficits. Orange indicates ORs >1, where thicker/larger structures are associated with greater social deficits. OCD: obsessive-compulsive disorder, ADHD: attention-deficit/ hyperactivity disorder, ASD: autism spectrum disorder, SCQ: Social Communication Questionnaire

Contrary to our initial hypotheses, the association between social brain region size and social deficits as measured by the SCQ varied significantly by diagnosis in the right lateral regions, frontal regions, left and right amygdala, hippocampus, bilateral dorsal striatum, and the left insula (*p* < 0.008 for all region-by-diagnosis interaction terms) (Table 2). Distinct patterns emerged for each diagnostic group (Fig. 1). In the control group, thinner lateral regions and insula, and smaller left amygdala and ventral striatal volumes were associated with higher SCQ scores, therefore greater social deficits (OR <1.0). Conversely, in the OCD group, while thinner cortical regions were associated with greater social deficits, *smaller* subcortical volumes and left insular thickness were associated with fewer social deficits (OR >1.0). In ADHD and ASD, thinner cortical and smaller subcortical structures were associated with increased social deficits in a similar pattern. Findings remained significant after FDR correction (Table 2). The distinct subcortical pattern (e.g., OR >1.0 in OCD, and <1.0 in ASD and ADHD) persisted after also adjusting for comorbid OCD symptoms (on the TOCS) or ADHD symptoms (on the CBCL-ADHD subscale) (data not shown). Across all findings, wide confidence intervals and significant spread on scatter plots (shown in Supplementary Figure 1) suggested a high degree of heterogeneity in patterns of association.

**Table 2:** Association between social regions of interest and social deficits on the SCQ

Region	Wald *Χ*^2^, *p* value		OR ASD, 95% CI	OR ADHD, 95% CI	OR OCD, 95% CI	OR Control, 95% CI
	Region	Region × Dx				
Lateral mentalization regions						
Left lateral regions	**12.21, 0.0005**		**0.55, 0.39–0.77**	**0.55, 0.39–0.77**	**0.55, 0.39–0.77**	**0.55, 0.39–0.77**
Right lateral regions		**16.62, 0.008**	0.71, 0.48–1.03	0.54, 0.27–1.07	**0.17, 0.083–0.33**	**0.12, 0.03–0.57**
Frontal cognitive regions						
Frontal regions		**18.85, 0.0003**	**0.49, 0.30–0.79**	0.44, 0.18–1.05	**0.03, 0.01–0.10**	0.29, 0.55–1.48
Deeper and subcortical affective regions						
Left amygdala^1^		**27.53,** <**0.0001**	**0.92, 0.87–0.98**	**0.79, 0.73–0.87**	**1.20, 1.03–1.41**	**0.60, 0.41–0.87**
Right amygdala^1^		**35.05**, <**0.0001**	1.01, 0.96–1.07	**0.84, 0.76–0.93**	**1.39, 1.21–1.61**	0.96, 0.87–1.07
Hippocampus^1^		**38.73**, <**0.0001**	**0.93, 0.88–0.97**	0.97, 0.89–1.06	**1.43, 1.25–1.63**	0.96, 0.84–1.10
Left dorsal striatum^2^		**32.97,** <**0.0001**	**0.88, 0.81–0.96**	**0.80, 0.68–0.93**	**1.72, 1.36–2.19**	0.99, 0.67–1.48
Right dorsal striatum^2^		**25.32,** <**0.0001**	**0.87, 0.80–0.95**	**0.80, 0.68–0.92**	**1.60, 1.24–2.05**	1.00, 0.70–1.43
Ventral striatum^2^	**14.86,** <**0.0001**		**0.63, 0.50–0.80**	**0.63, 0.50–0.80**	**0.63, 0.50–0.80**	**0.63, 0.50–0.80**
Left insula		**12.79, 0.005**	1.05, 0.75–1.46	1.41, 0.65–3.04	**6.71, 2.53–17.84**	1.40, 0.38–5.19
Right insula	**7.52, 0.006**		**0.70, 0.53–0.90**	**0.70, 0** **.53–0.90**	**0.70, 0.53–0.90**	**0.70, 0.53–0.90**

*Dx* diagnosis, *ASD* autism spectrum disorder, *ADHD* attention-deficit/hyperactivity disorder, *OCD* obsessive-compulsive disorder, *SCQ* Social Communication Questionnaire, social interaction, and communication items only, *OR* odds ratios, *FDR* false discovery rate

ORs are the odds of scoring on an SCQ item per mm increase in thickness, or ^1^0.1cm^3^ increase in volume, or ^2^1.0 cm increase in volume. Models treat SCQ scored/total as the dependent variable, and have been adjusted for the effects of age, sex, diagnosis, scanner upgrade, and diagnosis-by-region interactions, as well as whole-brain volume for volumetric structures. Where diagnosis-by-region interactions were non-significant (*p* > 0.05), they were dropped from the model. Bolded values remained significant (*q* < 0.05) after FDR correction

### Social perception deficits inversely correlated with amygdala and hippocampal volumes across diagnoses

**Fig. 2:**
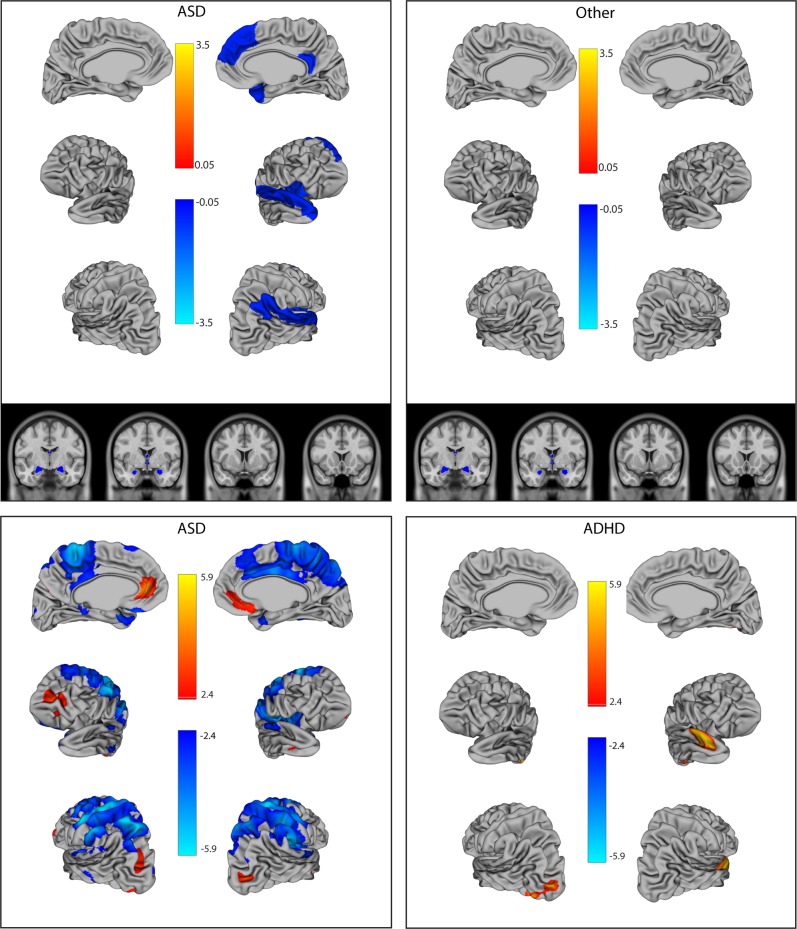
Log odds ratios of the association between brain structure and social perception deficits on the RMET. Top panels show the region of interest approach; log odds ratios (ORs) are for regions that were significant the multivariable model after false discovery rate (FDR) correction (*q* = 0.05). The bottom panels present the vertex-wise analysis for ASD and ADHD; vertices are shown that remained significant after an FDR correction at *q* = 0.05 (no vertices remained significant for the controls or OCD group). ORs indicate the change in RMET score per increase in size (per mm increase in thickness, 0.1 cm^3^ increase in volume for the amygdala/hippocampus, or 1.0 cm^3^ increase in volume for other subcortical structures). Blue indicates ORs <1, where thicker/larger structures are associated with fewer deficits. Orange indicates ORs >1, where thicker/larger structures are associated with greater social deficits. OCD: obsessive-compulsive disorder, ADHD: attention-deficit/ hyperactivity disorder, ASD: autism spectrum disorder, RMET: Reading the Mind in the Eyes Test

On the RMET, region-by-diagnosis interaction terms were only significant in the right and left lateral regions (*p* = 0.02 and 0.04) and the right insula (*p* = 0.02), although findings became non-significant after FDR correction. For these regions, interaction terms were kept in the model to convey patterns, however. Specifically, the ASD group showed a significant association (thinner cortex = greater deficits) and other groups were non-significant (Table 3, Fig. 2 (top panel)). Elsewhere, larger left amygdala volume (*p* < 0.0001), right amygdala volume (*p* = 0.003), and hippocampal volumes (*p* = 0.001) were associated with fewer social deficits across groups. As a sensitivity analysis, we also adjusted for IQ in the model, given data showing that RMET scores vary with cognition^1^. Adjusting for IQ resulted in a similar pattern of associations (Supplementary Table 4).

**Table 3:** Association between social regions of interest and social deficits on the RMET

Region	Wald *Χ*^2^, *p* value		OR ASD	OR ADHD	OR OCD	OR Control
	Region	Region × Dx				
Lateral mentalization regions						
Left lateral regions		8.16, *p* = 0.04	0.60, 0.36–0.98	1.34, 0.73–2.46	2.77, 0.72–10.61	1.42, 0.50–4.12
Right lateral regions		**9.51,** ***p*** = 0.02	**0.55, 0.35–0.86**	1.52, 0.86–2.68	1.24, 0.60–2.55	0.94, 0.34–2.65
Frontal cognitive regions						
Frontal regions	0.58, *p* = 0.4		0.85, 0.55–1.30	0.85, 0.55–1.30	0.85, 0.55–1.30	0.85, 0.55–1.30
Deeper and subcortical affective regions						
Left amygdala^1^	**23.34,** ***p*** < 0.0001		**0.88, 0.83–0.92**	**0.88, 0.83–0.92**	**0.88, 0.83–0.92**	**0.88, 0.83–0.92**
Right amygdala^1^	**8.74,** ***p*** = 0.003		**0.94, 0.90–0.98**	**0.94, 0.90–0.98**	**0.94, 0.90–0.98**	**0.94, 0.90–0.98**
Hippocampus^1^	**10.71,** ***p*** = 0.001		**0.93, 0.89–0.97**	**0.93, 0.89–0.97**	**0.93, 0.89–0.97**	**0.93, 0.89–0.97**
Left dorsal striatum^2^	0.05, *p* = 0.8		0.99, 0.92–1.07	0.99, 0.92–1.07	0.99, 0.92–1.07	0.99, 0.92–1.07
Right dorsal striatum^2^	0.00, *p* = 0.9		1.00, 0.92–1.08	1.00, 0.92–1.08	1.00, 0.92–1.08	1.00, 0.92–1.08
Ventral striatum^2^	0.47, *p* = 0.5		1.09, 0.86–1.38	1.09, 0.86–1.38	1.09, 0.86–1.38	1.09, 0.86–1.38
Left insula	0.58, *p* = 0.4		0.89, 0.65–1.21	0.89, 0.65–1.21	0.89, 0.65–1.21	0.89, 0.65–1.21
Right insula		**9.63,** ***p*** = 0.02	**0.48, 0.33–0** **.69**	1.21, 0.72–2.03	1.02, 0.48–2.17	0.61, 0.28–1.31

*Dx* diagnosis, *ASD* autism spectrum disorder, *ADHD*-attention-deficit/hyperactivity disorder, *OCD* obsessive-compulsive disorder, *RMET* Reading the Mind in the Eyes Test incorrect item, *OR* odds ratios

ORs are the odds of scoring incorrectly on an RMET item per mm increase in thickness, or ^1^0.1cm^3^ increase in volume, or ^2^1.0cm increase in volume. Models treat RMET incorrect/total as the dependent variable, and have been adjusted for the effects of age, sex, diagnosis, scanner upgrade, and diagnosis-by-region interactions, as well as whole-brain volume for volumetric structures. Where diagnosis-by-region interactions were non-significant (*p* > 0.05), they were dropped from the model. Bolded values remained significant (*q* < 0.05) after FDR correction

### Whole-brain vertex-wise analysis

**Fig. 3:**
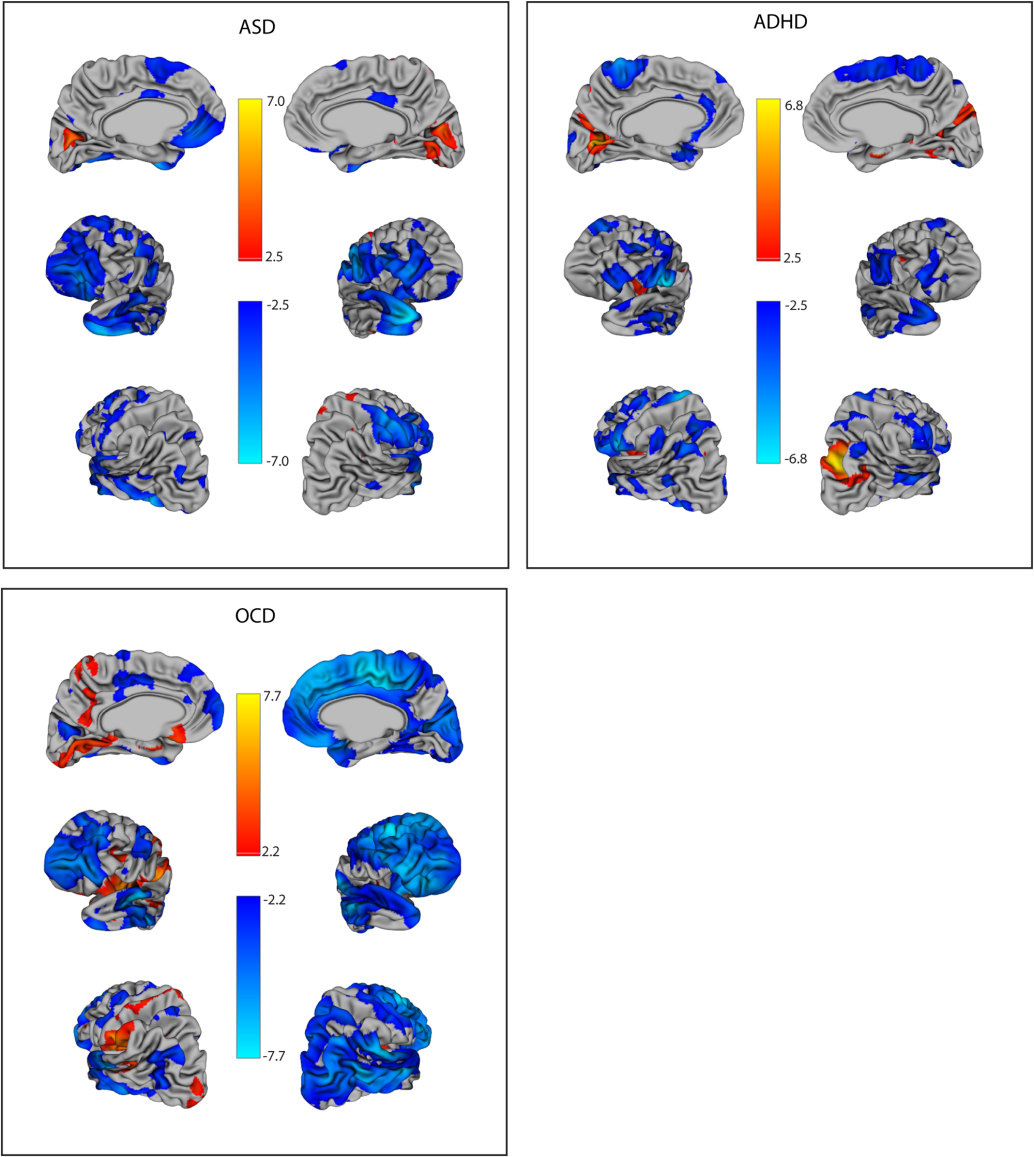
Log odds ratios of the association between cortical thickness and social communication deficits on the SCQ using a vertex-wise analysis. Vertices are shown that remained significant after an false discovery rate (FDR) correction at *q* = 0.05 (no vertices remained significant for the control group). Odds ratios (ORs) indicate the change in SCQ score per increase in size (per mm increase in thickness, 0.1 cm^3^ increase in volume for the amygdala/hippocampus, or 1.0 cm^3^ increase in volume for other subcortical structures). Blue indicates ORs <1, where thicker/larger structures are associated with fewer deficits. Orange indicates ORs >1, where thicker/larger structures are associated with greater social deficits. OCD: obsessive-compulsive disorder, ADHD: attention-deficit/hyperactivity disorder, ASD: autism spectrum disorder, SCQ: Social Communication Questionnaire

On whole-brain vertex-wise analysis, for the SCQ, patterns of associations were similar as found with the region of interest approach (Fig. 3, Supplementary Tables 5 and 6). Specifically, in the right lateral regions, 12% of voxels were significant in ADHD, 24% in ASD, and 87% in OCD, where generally thicker cortices were associated with fewer social deficits. For the left lateral regions, group differences were less pronounced (17% in ADHD, 22% in ASD, and 30% in OCD). Similarly, for the combined frontal regions, 18% of voxels were significant in ADHD, 39% in ASD, and 70% in OCD. The number of statistical peaks within a five vertex radius generally mirrored the proportion of significant voxels (Supplementary Tables 5 and 6). No associations remained significant after FDR correction for the control group. For OCD, vertex-wise associations were more lateralized than with the region of interest approach. The majority of the right hemisphere showed significant associations (where thicker cortex = fewer deficits); there were reciprocal effects (thicker cortex = greater social deficits) in the left insula (see Fig. 3). For the RMET, participants with ASD had fewer social deficits with thicker cortices in both the left (16% of voxels) and right (24% of voxels) lateral regions, with scattered frontal associations (30% of voxels), partially consistent with patterns in the region of interest analyses (Fig. 2, bottom panels). For the ADHD group, thicker cortices were associated with greater social deficits in the right temporal lobe, and the left occipital lobe. No regions remained significant after FDR correction for the OCD or control groups on the RMET.

### The association between brain structure and social communication abilities may vary by age

As an exploratory analysis, we also examined for age effects on the association between brain region and social deficits by checking for age-by-region interaction terms for the regions of interest. On the SCQ, the right lateral region showed a highly significant (*χ*^2^ = 21.82, *p* < 0.0001) age-by-region interaction. Specifically, the association between right lateral region thickness and SCQ score was more pronounced at younger ages (age 7 OR: 0.17 (0.10–0.30)), and was less significant in older children (age 11 OR: 0.39 (0.29–0.53), age 15 OR: 0.89 (0.57–1.35)). Several other regions showed a similar pattern, although the effect was less pronounced and would become non-significant after a correction for multiple comparisons (age-by-region interaction term *p* value for left lateral regions = 0.03, frontal regions = 0.02, hippocampus = 0.02, right dorsal striatum = 0.01). There were no significant age-by-region interactions for analyses involving the RMET. For the right lateral region, we then also tested for a three-way interaction term “age-by-region-by-diagnosis,” to determine whether the age effects on the brain–behavior relationship varied across diagnostic groups. There was a nominally significant three-way interaction term (*p* = 0.05). ASD and ADHD had more significant associations between brain and behavior on the SCQ at younger ages (OR age 7 for ASD: 0.20 (0.10–0.40); OR age 7 ADHD: 0.24 (0.08–0.70); OR age 15 ASD: 1.81 (1.07–3.06); OR age 15 ADHD: 0.94 (0.23–3.82)), whereas the opposite pattern was observed for the OCD and control groups (OR age 7 control: 0.24 (0.03–1.80), OR age 7 OCD: 0.50 (0.02–14.2); OR age 15 control: 0.02 (0.01–0.4), OR age 15 OCD (0.12 (0.03–0.45)).

### Exploratory analysis involving oxytocin receptor (*OXTR*) polymorphisms

There were 100 participants with ASD, and 51 participants with ADHD who also had genotype data available regarding single-nucleotide polymorphisms (SNPs) in the *OXTR* gene (Supplementary Table 7). Previous work on the same study sample suggested three SNPs in *OXTR* (*rs53576*, *rs237997*, and *rs2254298*) modify the severity of social deficits in ASD and ADHD^72^. We therefore examined for associations between *OXTR* genotype and brain structure in brain regions previously found to be affected by *OXTR* genotype in control populations (insula, limbic system, dorsal cingulate, temporal–parietal (lateral) regions)^73–80^. Exploratory analyses (described in Supplementary Methods) failed to detect any significant associations between *OXTR* genotype and brain structure after correction for multiple comparisons (Bonferroni correction with 24 tests, *p* = 0.002), with the exception of the left insula, in ADHD (Supplementary Table 8). Here, *rs53576* AA allele carriers had thicker cortices in the insula compared to other genotype groups (AA vs. GA *p* = 0.0005, AA vs. GG *p* = 0.0008).

## Discussion

In this study, we examined and compared the structural neuroimaging correlates of social abilities across a large sample of children and youth with different neurodevelopmental disorders. Overall data suggest similarities in the biological substrates of social communication abilities in ASD and ADHD; both had widely distributed associations between brain structure and SCQ score, involving multiple subcortical and cortical regions. Exploratory analyses identified age-associated difference in this effect in the right lateral regions, which were congruent in ASD and ADHD as well. For social perception on the RMET, all four groups showed a similar pattern of associations involving subcortical regions, while participants with ASD also had significant findings in the right insula and the right lateral regions. Across all diagnostic groups, associations were highly heterogeneous.

Our results are consistent with emerging data suggesting ASD and ADHD may be overlapping conditions, with related neuropathology and symptomatology^81,82^. For example, diffusion tensor imaging has shown a lack of diagnostic differences in fractional anisotropy of white matter tracts between ASD and ADHD, while OCD appeared distinct^8^. Latent class analysis of symptom surveys and cognitive profiles suggest overlapping cognitive deficits in both disorders^81^. Patterns of social perception deficits were strikingly similar in ASD and ADHD as well^1^. Rare copy number variants affecting many of the same genes have been shown to contribute to risk for both ASD and ADHD^83^.

Previous studies examining the association between brain structure and social deficits have yielded inconsistent results. Most studies have used the Social Responsiveness Scale (SRS) or subscales of the ADI-R to quantify social communication deficits, primarily in control populations. Recent work suggests the SRS may be highly sensitive to non-specific behavioral problems, reflecting general levels of impairment more so than social deficits per se^84^. In this study, we used the SCQ, which has been found to be a better screening instrument for ASD compared to the SRS^85^, and is not affected by IQ like other measures^85,86^. Despite different measures, our results using the SCQ (i.e., thicker cortices in frontal/temporal–parietal regions being associated with fewer deficits) are broadly consistent with several studies examining structural neuroimaging correlates of the SRS/ADI-R in control participants^87–89^ and in ASD^26,90^. The direction of our results are inconsistent with two larger studies, the first using the SRS in a large population of typically developing in 6–10 year olds^24^, and the second using the Autism Diagnostic Observation Schedule in ASD across the lifespan^29^. Difference in results may be explained in part by true biological differences in the neural substrates of these measures, heterogeneity across disorders and populations, variability in the age ranges of included participants, use of models that may or may not account for comorbid symptomatology/differences in IQ, and variation in the measurement aspects of the underlying social construct in different disorders.

Functional and structural neuroimaging work has associated RMET performance with activation/size of the inferior frontal gyrus, middle/posterior temporal regions, and the amygdala/hippocampus in typically developing adults^91–94^, although findings may defer in ASD^67,95^. Our results for the RMET highlight subcortical structures as being important across disorders; children with ASD also had associations in the lateral mentalization regions and in the insula. It is possible that this finding is reflective of more immature or inefficient social perception abilities in children with ASD, leading them to rely on additional cortical circuits to make social evaluations^96^.

Exploratory analyses identified age-associated differences in the association between right lateral region thickness and SCQ score; data also suggested that these age-associated differences may vary by diagnostic group. Existing literature supports the hypothesis that the neural substrates of social abilities may change over the course of development. For example, previous longitudinal work has shown that the patterns of functional MRI (fMRI) activation during facial/emotion recognition tasks change with age, and in particular, after puberty^97,98^. In control participants, completion of the RMET was associated with activation in the posterior superior temporal sulcus across age ranges, but younger children (with potentially more immature social perception abilities) also activated the prefrontal cortex, the inferior frontal gyrus, and the temporal pole during this task as well^96^. Given that the direction of the association between cortical thickness and general intelligence may change over childhood (from a negative correlation in younger children to a positive correlation in older children)^99^, and that neurodevelopmental disorders such as ASD and ADHD are associated with aberrant cortical maturation^15,18^, further research is merited in order to characterize disorder-specific longitudinal changes in the neural substrates of social deficits.

The pattern involving OCD on the SCQ is notably different with *larger* subcortical structures being associated with greater social impairments. Previous research has found frontal–subcortical hyperactivity during processing of emotional stimuli in OCD on fMRI^100^. Therefore, it is possible that the social deficits associated with increased subcortical volumes reflect aberrant processing of social–emotional information in OCD. Consistent with this hypothesis, the distinct OCD pattern in our data persisted even after adjusting for severity of OCD symptoms. The OCD group also showed strong lateralization effects with respect to the association between social abilities and insula thickness, consistent with previous data in control populations^70^. Overall, despite overlapping symptomatology with other disorders (in particular, repetitive behavior in ASD), the pattern of brain–behavior associations involving the OCD group appeared quite distinct.

The structural finding of nominally larger hippocampal volumes in OCD is consistent with data from a recent large meta-analysis showing a trend towards larger hippocampi (*p* = 0.09) in 107 pediatric OCD patients compared with 210 controls^21^. This stands in contrast to adult populations, where smaller hippocampi were found^21^. Notably, across other social brain regions of interest, there were no significant structural differences in thickness or volume between the four diagnostic groups. Meta-analyses comparing over 1000 participants with ASD^20^, or ADHD^23^, to control participants have found smaller subcortical volumes in both ASD and ADHD. Cortical differences have varied by study, and by age, for both disorders, however^18,20^. These results further highlight a lack of biological differences between ASD and ADHD in spite of differences in mean scores on the SCQ and RMET.

Oxytocin is a pituitary neuropeptide known to affect social behavior. A few small studies, in typically developing adult cohorts, have examined whether common genetic differences in the oxytocin receptor gene (*OXTR*) corresponded with differences in structural neuroanatomy^77–80,101^. Specifically, different *OXTR* rs2254298 and rs53576 genotypes corresponded with differences in amygdala volumes^77,78,80^, and *OXTR* rs2254298 genotype was associated with differences in thickness of the right insula and dorsal anterior cingulate as well^79,101^. Our exploratory analyses did not reproduce these findings in children with neurodevelopmental disorders, although rs53576 genotype was associated with thickness in the left insula in the ADHD group. Despite this, overall trends suggested a similar direction of gene–brain associations in ASD and ADHD.

In terms of limitations, sample sizes were unequal between groups, contributing to less confidence to detect differences in the OCD and control groups. Regions were grouped anatomically and functionally to facilitate description of overall patterns; consensus has yet to be reached with the respect to the specific regions and functions of social neural networks. The SCQ is a parent report measure that captures complex aspects of social communication (making friends, using non-verbal communication, interest in others); diagnostic differences in the measurement aspects of this measure were not the focus of the paper but could also contribute to results. SCQ scores were right skewed in controls, ADHD, and OCD, but left skewed in ASD, raising the possibility of influential outliers driving results. Case-wise diagnostics were conducted and repeat analyses excluding 13 potential influential outliers based on difchi-squared values yielded the same pattern of findings (note no dfbetas were >2). Restricting the range of SCQ scores to 0–15 resulted in similar patterns as well, although less significant results. The group with ASD tended to be higher functioning; results may be less generalizable to a more severely affected population. Also the oxytocin results should be considered exploratory in view of the small sample size and the multiple testing.

Overall, this study revealed that the structural neuroimaging correlates of social communication and social perception deficits vary across neurodevelopmental disorders. There were more similarities than differences between ASD and ADHD, even on an ASD-specific outcome measure. Data support a common underlying biology and the blurring of the diagnostic boundaries between these two disorders.

## Supplementary information

SUPPLEMENTARY INFORMATION
