# Supplementary material for: Structural neuroimaging correlates of social deficits are similar in autism spectrum disorder and attention-deficit/hyperactivity disorder: analysis from the POND Network
Source: Transl Psychiatry. 2019 Feb 4;9:72. doi: 10.1038/s41398-019-0382-0 (PMC6361977; doi:10.1038/s41398-019-0382-0)
Supplement: Supplementary file 1 — SUPPLEMENTARY INFORMATION [file 41398_2019_382_MOESM1_ESM.docx]

**SUPPLEMENTARY INFORMATION**

**Structural Neuroimaging Correlates of Social Deficits are Similar in Autism Spectrum Disorder and Attention- Deficit/Hyperactivity Disorder: Analysis from the POND Network**

Danielle A. Baribeau, M.D., Annie Dupuis, Ph.D., Tara A. Paton, Ph.D., Christopher Hammill, M.Sc.Stephen W. Scherer, Ph.D., Russell J. Schachar, M.D., Paul D. Arnold, M.D., Ph.D., Peter Szatmari, M.D., Rob Nicolson, M.D., Stelios Georgiades, Ph.D., Jennifer Crosbie, Ph.D., Jessica Brian, Ph.D., Alana Iaboni, Ph.D., Azadeh Kushki, Ph.D., Jason Lerch, Ph.D., Evdokia Anagnostou, M.D.

**CONTENTS:**

1. Supplementary Table 1: Brain Regions Involved in Processing Social Information (Page 2)
2. Supplementary Table 2: Pearson Correlation Coefficients between Behavioral Measures (page 3)
3. Supplementary Table 3: Least Squares Mean Thickness/ Volume by Region of Interest per Diagnostic Group (Page 4)
4. Supplementary Table 4: Association Between Social Regions and Social Deficits on the RMET adjusting for IQ (Page 5)
5. Supplementary Figure 1: Scatter plots of SCQ/ RMET scores and Brain thickness/volume in Social Brain Regions (Page 6)
6. Supplementary Table 5: Number of Significant Voxels per Region on the Vertex-Wise Analysis (Page 7-9)
7. Supplementary Table 6: Number of Peak Intensities per Region of Interest (Page 10-11)
8. Supplementary Table 7: *OXTR* Allele Frequencies (Page 12)
9. Supplementary Methods for Exploratory Analyses involving *OXTR* (Page 13)
10. Supplementary Table 6: The Association Between *OXTR* genotype and Brain Structure in ASD and ADHD (Page 14-15)
11. References (Page 16)

**Supplementary Table 1: Brain Regions Involved in Processing Social Information**

| **Summary of Reviews/ Meta-analyses describing Models of the Social Brain** | | | | |
| --- | --- | --- | --- | --- |
| **Reference** | | **Method** | **Social Model/ Process (Regions)** | |
| (18) | | Meta-analysis of fMRI studies | (1) Lower sensory (fusiform gyrus, pSTS, middle temporal), (2) Limbic (amygdala, hippocampus, nucleus accumbens, rostral ACC, vmPFC), (3) Intermediate (mid cingulate, anterior insula, IFG, supramarginal gyrus, cerebellum) and (4) High associative neural circuits (dmPFC, frontal poles, temporal poles, posterior cingulate, precuneus, middle temporal gyrus, TPJ) | |
| (17) | | Narrative review and proposed theoretical model | (1) Cognitive control (dorsal and ventral lateral PFC, posterior medial PFC, dorsal ACC); (2) Empathy/ social cognition (dorsal premotor regions, TPJ, dmPFC, precuneus); (3) Affective responding (insula, amygdala, ventral striatum) | |
| (15, 16) | | Narrative review and proposed theoretical model | (1) Amygdala network (e.g., amygdala, temporal regions, dmPFC, OFC; (2) Mentalizing network (e.g., temporal poles, TPJ, STS/STG, posterior cingulate; (3) Empathy network (e.g., insula, dorsal cingulate) (4) Mirror/ simulation network | |
| (19) | | Review of lesion studies and proposed theoretical model | (1) Emotional empathy (amygdala, temporal pole, anterior insula, ACC (2) Cognitive perspective taking (left medial PFC); (3) Mentalizing (TPJ); (4) Affective perspective taking (right medial PFC) (5) Emotional contagion (OFC, inferior frontal cortex) | |
| (20) | | Meta-analysis of fMRI studies | (1) Social judgement and trait inferences (Right TPJ and mPFC) | |
| **Simplified Synthesis of Social Brain Regions** | | | | |
|  | **Midline and lateral regions (group 1)** | | **Anterior and prefrontal regions (group 2)** | **Deeper cortical and subcortical structures (group 3)** |
| Function | Mentalization, empathy, and processing of visual sensory cues | | Executive function and cognitive control over affective and social processes | Affective responding, memory, and social reward processing |
| Regions | - TPJ  - STS/STG  - dmPFC  - Temporal poles  - Posterior cingulate | | - ACC  - Dorsal cingulate  - OFC  - dlPFC  - vlPFC | - Insula  - Amygdala  - Hippocampus  - Dorsal striatum  - Ventral striatum |

pSTS: posterior superior temporal sulcus; STS/STG: superior temporal gyrus/ sulcus; ACC: anterior cingulate cortex; TPJ: temporal parietal junction; dmPFC: dorsal medial prefrontal cortex; dlPFC: dorsal lateral prefrontal cortex; vlPFC: ventral lateral prefrontal cortex; OFC: orbital frontal cortex.

**Supplementary Table 2: Spearman Correlation Coefficients between Behavioral Measures**

|  | **Spearman Correlation Coefficients** p-value Number of Observations | | | | | |
| --- | --- | --- | --- | --- | --- | --- |
|  | **SCQ Total** | **SCQ**  **Soc Com** | **RMET** | **CBCL-**  **ADHD** | **IQ** | **TOCS** |
| **SCQ Total** | 1.00000    294 | 0.96488  <.0001  293 | -0.24952  0.0001  234 | 0.39328  <.0001  282 | -0.41665  <.0001  241 | 0.08165  0.1877  262 |
| **SCQ Soc Com** | 0.96488  <.0001  293 | 1.00000    293 | -0.21597  0.0009  233 | 0.33946  <.0001  281 | -0.43751  <.0001  240 | 0.03114  0.6166  261 |
| **RMET** | -0.24952  0.0001  234 | -0.21597  0.0009  233 | 1.00000    241 | 0.01397  0.8327  231 | 0.45915  <.0001  208 | 0.24559  0.0003  215 |
| **CBCL-ADHD** | 0.39328  <.0001  282 | 0.33946  <.0001  281 | 0.01397  0.8327  231 | 1.00000    284 | -0.16120  0.0140  232 | 0.46152  <.0001  259 |
| **IQ** | -0.41665  <.0001  241 | -0.43751  <.0001  240 | 0.45915  <.0001  208 | -0.16120  0.0140  232 | 1.00000    249 | 0.18134  0.0080  213 |
| **TOCS** | 0.08165  0.1877  262 | 0.03114  0.6166  261 | 0.24559  0.0003  215 | 0.46152  <.0001  259 | 0.18134  0.0080  213 | 1.00000    264 |

SCQ: Social Communication Questionnaire (n=39 items). SCQ Soc Com: Social Communication Questionnaire Social and Communication items only (n= 28 items). IQ: Intelligence Quotient. RMET: Reading the Mind in the Eyes Task number of correct items out of 28. CBCL-ADHD: Child Behavior Checklist ages 6-18 ADHD Subscale T-score. TOCS: Toronto Obsessive-Compulsive Scale. Some participants had missing data for various measures; the number of participants with data available is presented in each cell

**Supplementary Table 3: Least Squares Mean Thickness/ Volume by Region of Interest per Diagnostic Group**

|  | **F dx** | **p-value** | **DX** | **LS mean** | **Lower CL** | **Upper**  **CL** | **P Pairwise** |
| --- | --- | --- | --- | --- | --- | --- | --- |
| whole brain thickness (mm) | 1.11 | 0.3 | **Control** | 3.73 | 3.68 | 3.77 |  |
|  |  |  | **OCD** | 3.69 | 3.66 | 3.73 |  |
|  |  |  | **ADHD** | 3.70 | 3.67 | 3.73 |  |
|  |  |  | **ASD** | 3.72 | 3.70 | 3.74 |  |
| whole brain volume (cm^3^) | 2.26 | 0.08 | **Control** | 1436.72 | 1395.34 | 1478.11 |  |
|  |  |  | **OCD** | 1470.09 | 1435.27 | 1504.90 |  |
|  |  |  | **ADHD** | 1412.73 | 1384.31 | 1441.15 |  |
|  |  |  | **ASD** | 1437.48 | 1416.93 | 1458.03 |  |
| combined frontal thickness (mm) | 0.22 | 0.9 | **Control** | 3.75 | 3.70 | 3.79 |  |
|  |  |  | **OCD** | 3.72 | 3.69 | 3.76 |  |
|  |  |  | **ADHD** | 3.73 | 3.70 | 3.77 |  |
|  |  |  | **ASD** | 3.74 | 3.71 | 3.79 |  |
| left lateral group thickness (mm) | 0.55 | 0.7 | **Control** | 4.02 | 3.97 | 4.08 |  |
|  |  |  | **OCD** | 4.03 | 3.98 | 4.08 |  |
|  |  |  | **ADHD** | 4.03 | 3.99 | 4.06 |  |
|  |  |  | **ASD** | 4.05 | 4.02 | 4.08 |  |
| right lateral group thickness (mm) | 1.78 | 0.2 | **Control** | 4.08 | 4.02 | 4.14 |  |
|  |  |  | **OCD** | 4.05 | 4.00 | 4.10 |  |
|  |  |  | **ADHD** | 4.07 | 4.03 | 4.11 |  |
|  |  |  | **ASD** | 4.12 | 4.08 | 4.14 |  |
| left amygdala volume (mm^3^) | 2.01 | 0.1 | **Control** | 1177.87 | 1128.30 | 1227.45 |  |
|  |  |  | **OCD** | 1218.54 | 1178.58 | 1258.51 |  |
|  |  |  | **ADHD** | 1168.59 | 1135.10 | 1202.08 |  |
|  |  |  | **ASD** | 1163.61 | 1140.35 | 1186.87 |  |
| right amygdala volume (mm^3^) | 2.08 | 0.1 | **Control** | 1153.60 | 1098.89 | 1208.31 |  |
|  |  |  | **OCD** | 1237.20 | 1192.45 | 1281.95 |  |
|  |  |  | **ADHD** | 1178.99 | 1141.55 | 1216.43 |  |
|  |  |  | **ASD** | 1188.22 | 1162.20 | 1214.25 |  |
| hippocampal volume (mm^3^)* | 4.10 | 0.007 | **Control** | 1158.98 | 1104.88 | 1213.08 | Vs. OCD=.05 |
|  |  |  | **OCD** | 1229.93 | 1185.67 | 1274.18 |  |
|  |  |  | **ADHD** | 1133.02 | 1096.00 | 1170.05 | Vs. OCD=.0007 |
|  |  |  | **ASD** | 1154.93 | 1129.19 | 1180.66 | Vs. OCD=.003 |
| left dorsal striatal volume (mm^3^) | 2.23 | 0.09 | **Control** | 8650.73 | 8324.97 | 8976.49 |  |
|  |  |  | **OCD** | 8988.13 | 8721.29 | 9254.97 |  |
|  |  |  | **ADHD** | 8570.76 | 8352.48 | 8789.03 |  |
|  |  |  | **ASD** | 8638.12 | 8483.25 | 8792.98 |  |
| right dorsal striatal volume (mm^3^) | 1.54 | 0.2 | **Control** | 8563.48 | 8233.55 | 8893.41 |  |
|  |  |  | **OCD** | 8841.86 | 8571.60 | 9112.11 |  |
|  |  |  | **ADHD** | 8490.30 | 8269.24 | 8711.37 |  |
|  |  |  | **ASD** | 8546.54 | 8389.69 | 8703.39 |  |
| ventral striatal volume (mm^3^) | 2.29 | 0.08 | **Control** | 2271.45 | 2167.70 | 2375.19 |  |
|  |  |  | **OCD** | 2321.40 | 2236.42 | 2406.39 |  |
|  |  |  | **ADHD** | 2201.76 | 2132.24 | 2271.27 |  |
|  |  |  | ASD | 2207.85 | 2158.52 | 2257.17 |  |
| Left Insula (mm) | 1.28 | 0.3 | **Control** | 4.14 | 4.08 | 4.21 |  |
|  |  |  | **OCD** | 4.19 | 4.14 | 4.24 |  |
|  |  |  | **ADHD** | 4.14 | 4.10 | 4.17 |  |
|  |  |  | **ASD** | 4.18 | 4.15 | 4.21 |  |
| Right Insula (mm) | 1.38 | 0.2 | **Control** | 4.23 | 4.15 | 4.30 |  |
|  |  |  | **OCD** | 4.21 | 4.15 | 4.27 |  |
|  |  |  | **ADHD** | 4.22 | 4.17 | 4.27 |  |
|  |  |  | **ASD** | 4.26 | 4.23 | 4.30 |  |

Least squares (LS) mean estimates for brain region by diagnostic group was calculated including age and sex as covariates in the model. *Also adjusting for whole brain volume in the model yielded a less significant difference for the hippocampus [F=2.62, p=0.05, Control= 1173.48 mm^3^ (1124.73-1222.22), OCD= 1236.56 mm^3^ (1197.29-1275.83), ADHD=1169.07 mm^3^ (1134.80-1203.33), ASD=1182.00 mm^3^ (1158.38-1205.62)]. All other volumetric structures remained non-significantly different by diagnosis after also adjusting for whole brain volume. CL= 95% confidence limit.

**Supplementary Table 4: Association Between Social Regions and Social Deficits on the RMET also adjusting for IQ**

| **Region** | **Wald** Χ^2^**, p-value** | | **OR ASD** | **OR ADHD** | **OR OCD** | **OR Control** |
| --- | --- | --- | --- | --- | --- | --- |
|  | **Region** | **Region× Dx** |  |  |  |  |
| **Lateral mentalization regions** | | |  |  |  |  |
| Left lateral regions | 0.22  p=0.6 |  | 0.91  0.62-1.34 | 0.91  0.62-1.34 | 0.91  0.62-1.34 | 0.91  0.62-1.34 |
| Right lateral regions |  | **13.32**  **p=0.004** | **0.53**  **0.33-0.85** | 2.03  1.09-3.79 | 1.30  0.58-2.93 | 0.77  0.27-2.21 |
| **Frontal cognitive regions** | | |  |  |  |  |
| Frontal Regions | 0.04  p=0.8 |  | 1.05  0.66-1.66 | 1.05  0.66-1.66 | 1.05  0.66-1.66 | 1.05  0.66-1.66 |
| **Deeper and subcortical affective regions** | | |  |  |  |  |
| Left amygdala* | **12.00**  **p=0.0005** |  | **0.90**  **0.85-0.96** | **0.90**  **0.85-0.96** | **0.90**  **0.85-0.96** | **0.90**  **0.85-0.96** |
| Right Amygdala* | **6.55**  **p=0.01** |  | **0.95**  **0.90-0.99** | **0.95**  **0.90-0.99** | **0.95**  **0.90-0.99** | **0.95**  **0.90-0.99** |
| Hippocampus* | **13.13**  **0.0003** |  | **0.93**  **0.89-0.97** | **0.93**  **0.89-0.97** | **0.93**  **0.89-0.97** | **0.93**  **0.89-0.97** |
| Left dorsal striatum** | 1.73  p=0.2 |  | 0.94  0.87-1.03 | 0.94  0.87-1.03 | 0.94  0.87-1.03 | 0.94  0.87-1.03 |
| Right dorsal striatum** | 1.44  p=0.2 |  | 0.95  0.87-1.03 | 0.95  0.87-1.03 | 0.95  0.87-1.03 | 0.95  0.87-1.03 |
| Ventral striatum** | 0.67  p=0.4 |  | 0.90  0.69-1.17 | 0.90  0.69-1.17 | 0.90  0.69-1.17 | 0.90  0.69-1.17 |
| Left Insula | 0.74  p=0.4 |  | 0.86  0.61-1.22 | 0.86  0.61-1.22 | 0.86  0.61-1.22 | 0.86  0.61-1.22 |
| Right Insula |  | **9.63**  **p=0.02** | **0.48**  **0.33-0.70** | 1.21  0.72-2.03 | 1.02  0.48-2.17 | 0.61  0.28-1.31 |

Dx: diagnosis, ASD: autism spectrum disorder, ADHD: attention-deficit/ hyperactivity disorder, OCD: obsessive-compulsive disorder, RMET: reading the mind in the eyes test incorrect item. Odds ratios (OR) for volumetric structures are the odds of scoring incorrectly on an RMET item per mm increase in thickness, or ^1^0.1 cm3 increase in volume, or ^2^1.0cm increase in volume. Models treat RMET incorrect/total as the dependent variable, and have been adjusted for the effects of age, sex, diagnosis, IQ, scanner upgrade, diagnosis-by-region interactions, as well as whole brain volume for volumetric structures. Where diagnosis-by-region interactions were non-significant (p>0.05), they were dropped from the model. Bolded values remained significant after FDR correction.

**Supplementary Figure 1: Scatter plots of SCQ/ RMET scores and Brain thickness/volume in Social Brain Regions** (Only regions with significant effects in the main multivariable models shown)

Dx: diagnosis, ASD: autism spectrum disorder, ADHD: attention-deficit/ hyperactivity disorder, OCD: obsessive-compulsive disorder, RMET: reading the mind in the eyes test incorrect item. SCQ: Social communication questionnaire, social/communication items only. Here, the unit of measurement for cortical thickness is mm, for subcortical volume is mm^3^. Scatter plots and best fit lines are shown for: 1) regions where the main effect of thickness/volume on social deficits was significant after FDR correction in the multivariable model, or 2) regions where the region-by-diagnosis interaction term was significant (p<0.05), for which the best fit line for all four diagnostic groups is displayed. Note, these are raw data only; multivariable models account for age, sex, scanner upgrade, and whole brain volume for volumetric structures as well.

**Supplementary Table 5: Number of Significant Voxels per Region on the Vertex-Wise Analysis**

| **Region of Interest** | **Included Structures** | **Total Voxel Count** | **ADHD SCQ** | **ASD SCQ** | **OCD SCQ** | **ADHD RMET** | **ASD RMET** |
| --- | --- | --- | --- | --- | --- | --- | --- |
| Right Lateral Mentalization Regions | Right Angular gyrus | 938 | 102 | 89 | 674 | 0 | 174 |
|  | Right Superior temporal gyrus | 1900 | 56 | 604 | 1624 | 291 | 526 |
|  | Right Superior frontal gyrus, medial | 1256 | 214 | 37 | 1256 | 0 | 458 |
|  | Right Temporal pole: middle temporal gyrus | 252 | 114 | 152 | 223 | 16 | 5 |
|  | Right Temporal pole: superior temporal gyrus | 656 | 164 | 389 | 656 | 121 | 62 |
|  | Right Posterior cingulate gyrus | 306 | 0 | 5 | 179 | 0 | 29 |
|  | Proportion of Significant Voxels | 5308 | 0.12 | 0.24 | 0.87 | 0.08 | 0.24 |
|  |  |  |  |  |  |  |  |
| Left Lateral Mentalization Regions | Left Angular gyrus | 938 | 308 | 60 | 186 | 0 | 159 |
|  | Left Superior temporal gyrus | 1900 | 34 | 403 | 778 | 0 | 168 |
|  | Left Superior frontal gyrus, medial | 1256 | 160 | 360 | 402 | 0 | 279 |
|  | Left Temporal pole: middle temporal gyrus | 252 | 1 | 232 | 108 | 0 | 5 |
|  | Left Temporal pole: superior temporal gyrus | 656 | 388 | 104 | 33 | 0 | 148 |
|  | Left Posterior cingulate gyrus | 306 | 0 | 0 | 74 | 0 | 82 |
|  | Proportion of Significant Voxels | 5308 | 0.17 | 0.22 | 0.30 | 0 | 0.16 |
|  |  |  |  |  |  |  |  |
| Frontal Cognitive Regions | Left Anterior cingulate and paracingulate gyri | 548 | 271 | 124 | 79 | 0 | 213 |
|  | Left Median cingulate and paracingulate gyri | 841 | 24 | 186 | 349 | 0 | 432 |
|  | Left Superior frontal gyrus, medial orbital | 471 | 0 | 360 | 234 | 0 | 136 |
|  | Left Middle frontal gyrus orbital part | 377 | 61 | 377 | 372 | 0 | 166 |
|  | Left Inferior frontal gyrus, orbital part | 771 | 343 | 564 | 353 | 0 | 48 |
|  | Left Superior frontal gyrus, medial orbital | 471 | 0 | 360 | 234 | 0 | 136 |
|  | Left Gyrus Rectus | 472 | 0 | 377 | 184 | 0 | 205 |
|  | Left Superior frontal gyrus, dorsolateral | 1722 | 304 | 925 | 824 | 0 | 1091 |
|  | Left Middle frontal gyrus | 2213 | 678 | 1071 | 1075 | 0 | 784 |
|  | Left Inferior frontal gyrus, opercular part | 468 | 217 | 83 | 17 | 0 | 0 |
|  | Left Inferior frontal gyrus, triangular part | 866 | 447 | 36 | 155 | 0 | 0 |
|  | Right Anterior cingulate and paracingulate gyri | 548 | 0 | 0 | 547 | 0 | 241 |
|  | Right Median cingulate and paracingulate gyri | 841 | 0 | 213 | 835 | 0 | 662 |
|  | Right Supeiror frontal gyrus, orbital part | 944 | 0 | 344 | 944 | 0 | 67 |
|  | Right Middle frontal gyrus orbital part | 377 | 72 | 168 | 377 | 0 | 0 |
|  | Right Inferior frontal gyrus, orbital part | 771 | 374 | 445 | 771 | 0 | 0 |
|  | Right Superior frontal gyrus, medial orbital | 471 | 0 | 0 | 471 | 0 | 175 |
|  | Right Gyrus Rectus | 472 | 0 | 249 | 472 | 0 | 36 |
|  | Right Superior frontal gyrus, dorsolateral | 1722 | 344 | 144 | 1676 | 0 | 973 |
|  | Right Middle frontal gyrus | 2213 | 116 | 598 | 2213 | 0 | 419 |
|  | Right Inferior frontal gyrus, opercular part | 468 | 0 | 223 | 253 | 0 | 36 |
|  | Right Inferior frontal gyrus, triangular part | 866 | 104 | 552 | 852 | 0 | 0 |
|  | Proportion of Significant Voxels | 18913 | 0.18 | 0.39 | 0.70 | 0 | 0.31 |
| **Region of Interest** | **Included Structures** | **Total Voxel Count** | **ADHD SCQ** | **ASD SCQ** | **OCD SCQ** | **ADHD RMET** | **ASD RMET** |
| Deeper Cortical Regions | Right Insula | 929 | 31 | 211 | 555 | 0 | 635 |
|  | Left Insula | 929 | 305 | 82 | 659 | 0 | 0 |
|  |  |  |  |  |  |  |  |
| Other Regions | Left Calcarine fissure and surrounding cortex | 1300 | 474 | 269 | 432 | 0 | 63 |
|  | Left Cuneus | 1127 | 510 | 179 | 200 | 0 | 27 |
|  | Left Fusiform gyrus | 1304 | 266 | 682 | 587 | 0 | 4 |
|  | Left Heschl gyrus | 305 | 0 | 61 | 164 | 0 | 76 |
|  | Left Inferior occipital gyrus | 551 | 227 | 315 | 110 | 157 | 95 |
|  | Left Inferior parietal, but supramarginal and angular gyri | 0 | 0 | 0 | 0 | 0 | 0 |
|  | Left Inferior temporal gyrus | 1256 | 492 | 767 | 1099 | 0 | 83 |
|  | Left Lingual gyrus | 929 | 161 | 209 | 425 | 0 | 48 |
|  | Left Middle occipital gyrus | 1404 | 147 | 146 | 565 | 104 | 85 |
|  | Left Middle temporal gyrus | 1898 | 596 | 955 | 770 | 0 | 27 |
|  | Left Olfactory Cortex | 114 | 51 | 109 | 109 | 0 | 61 |
|  | Left Paracentral lobule | 1064 | 729 | 6 | 229 | 0 | 995 |
|  | Left Parahippocampal gyrus | 772 | 54 | 105 | 415 | 0 | 32 |
|  | Left Postcentral gyrus | 2123 | 621 | 238 | 825 | 0 | 1331 |
|  | Left Precentral gyrus | 1880 | 1062 | 733 | 921 | 0 | 1436 |
|  | Left Precuneus | 1532 | 10 | 58 | 294 | 0 | 175 |
|  | Left Rolandic operculum | 606 | 226 | 0 | 198 | 0 | 0 |
|  | Left Supeiror frontal gyrus, orbital part | 944 | 3 | 805 | 808 | 0 | 298 |
|  | Left Superior occipital gyrus | 840 | 17 | 62 | 95 | 93 | 217 |
|  | Left Superior parietal gyrus | 1417 | 302 | 88 | 202 | 0 | 1009 |
|  | Left Supplementary motor area | 968 | 59 | 274 | 246 | 0 | 270 |
|  | Left Supramarginal gyrus | 1135 | 181 | 6 | 543 | 0 | 603 |
|  | Right Calcarine fissure and surrounding cortex | 1300 | 134 | 222 | 784 | 0 | 0 |
|  | Right Cuneus | 1127 | 394 | 201 | 1127 | 0 | 164 |
|  | Right Fusiform gyrus | 1304 | 91 | 175 | 555 | 34 | 105 |
|  | Right Heschl gyrus | 305 | 0 | 84 | 100 | 0 | 258 |
|  | Right Inferior occipital gyrus | 551 | 54 | 8 | 213 | 5 | 0 |
|  | Right Inferior parietal, but supramarginal and angular gyri | 0 | 0 | 0 | 0 | 0 | 0 |
|  | Right Inferior temporal gyrus | 1256 | 87 | 311 | 724 | 0 | 17 |
|  | Right Lingual gyrus | 929 | 260 | 197 | 608 | 31 | 0 |
|  | Right Middle frontal gyrus | 2213 | 116 | 598 | 2213 | 0 | 419 |
|  | Right Middle occipital gyrus | 1404 | 371 | 33 | 1334 | 0 | 113 |
|  | Right Middle temporal gyrus | 1898 | 652 | 358 | 1419 | 98 | 0 |
|  | Right Olfactory Cortex | 114 | 1 | 54 | 108 | 0 | 55 |
|  | Right Paracentral lobule | 1064 | 584 | 7 | 910 | 0 | 1008 |
|  | Right Parahippocampal gyrus | 772 | 39 | 150 | 138 | 0 | 23 |
|  | Right Postcentral gyrus | 2123 | 739 | 1147 | 1089 | 0 | 1447 |
| **Region of Interest** | **Included Structures** | **Total Voxel Count** | **ADHD SCQ** | **ASD SCQ** | **OCD SCQ** | **ADHD RMET** | **ASD RMET** |
| Other Regions Continued | Right Precentral gyrus | 1880 | 633 | 962 | 1565 | 0 | 1243 |
|  | Right Precuneus | 1532 | 80 | 20 | 958 | 0 | 872 |
|  | Right Rolandic operculum | 606 | 332 | 145 | 0 | 0 | 309 |
|  | Right Superior frontal gyrus, dorsolateral | 1722 | 344 | 144 | 1676 | 0 | 973 |
|  | Right Superior occipital gyrus | 840 | 549 | 0 | 690 | 0 | 127 |
|  | Right Superior parietal gyrus | 1417 | 431 | 41 | 1097 | 0 | 933 |
|  | Right Supplementary motor area | 968 | 582 | 62 | 968 | 0 | 617 |
|  | Right Supramarginal gyrus | 1135 | 118 | 413 | 166 | 0 | 1017 |

ASD: autism spectrum disorder, ADHD: attention-deficit/ hyperactivity disorder, OCD: obsessive-compulsive disorder, RMET: reading the mind in the eyes test incorrect item. SCQ: Social communication questionnaire, social/communication items only.

**Supplementary Table 6: Number of Peak Intensities per Region of Interest**

| Region of Interest | Included Structures | **ADHD SCQ** | **ASD SCQ** | **OCD SCQ** | **ADHD RMET** | **ASD RMET** |
| --- | --- | --- | --- | --- | --- | --- |
| Right Lateral Mentalization Regions | | | | | | |
|  | Right Angular gyrus | 2 | 2 | 3 | 0 | 0 |
|  | Right Superior temporal gyrus | 1 | 2 | 4 | 1 | 1 |
|  | Right Superior frontal gyrus, medial | 0 | 0 | 4 | 0 | 2 |
|  | Right Temporal pole: middle temporal gyrus | 1 | 1 | 0 | 0 | 0 |
|  | Right Temporal pole: superior temporal gyrus | 1 | 0 | 2 | 1 | 1 |
|  | Right Posterior cingulate gyrus | 0 | 0 | 0 | 0 | 0 |
|  | Total | 5 | 5 | 13 | 2 | 4 |
|  |  |  |  |  |  |  |
| Left Lateral Mentalization Regions | | | | | | |
|  | Left Angular gyrus | 1 | 2 | 2 | 0 | 1 |
|  | Left Superior temporal gyrus | 0 | 2 | 2 | 0 | 2 |
|  | Left Superior frontal gyrus, medial | 0 | 0 | 0 | 0 | 0 |
|  | Left Temporal pole: middle temporal gyrus | 0 | 0 | 1 | 0 | 0 |
|  | Left Temporal pole: superior temporal gyrus | 3 | 0 | 1 | 0 | 2 |
|  | Left Posterior cingulate gyrus | 0 | 0 | 1 | 0 | 0 |
|  | Total | 4 | 4 | 7 | 0 | 5 |
|  |  |  |  |  |  |  |
| Frontal Cognitive Regions | | | | | | |
|  | Left Anterior cingulate and paracingulate gyri | 2 | 1 | 2 | 0 | 1 |
|  | Left Median cingulate and paracingulate gyri | 1 | 2 | 4 | 0 | 2 |
|  | Left Supeiror frontal gyrus, orbital part | 0 | 3 | 0 | 0 | 2 |
|  | Left Middle frontal gyrus orbital part | 0 | 1 | 2 | 0 | 2 |
|  | Left Inferior frontal gyrus, orbital part | 3 | 3 | 1 | 0 | 0 |
|  | Left Superior frontal gyrus, medial orbital | 0 | 1 | 0 | 0 | 0 |
|  | Left Gyrus Rectus | 0 | 0 | 1 | 0 | 2 |
|  | Left Superior frontal gyrus, dorsolateral | 2 | 4 | 2 | 0 | 3 |
|  | Left Middle frontal gyrus | 3 | 6 | 5 | 0 | 2 |
|  | Left Inferior frontal gyrus, opercular part | 1 | 1 | 1 | 0 | 0 |
|  | Left Inferior frontal gyrus, triangular part | 0 | 0 | 1 | 0 | 0 |
|  | Right Anterior cingulate and paracingulate gyri | 0 | 0 | 1 | 0 | 2 |
|  | Right Median cingulate and paracingulate gyri | 0 | 2 | 1 | 0 | 3 |
|  | Right Supeiror frontal gyrus, orbital part | 0 | 3 | 2 | 0 | 1 |
|  | Right Middle frontal gyrus orbital part | 0 | 0 | 0 | 0 | 0 |
|  | Right Inferior frontal gyrus, orbital part | 4 | 1 | 1 | 0 | 0 |
|  | Right Superior frontal gyrus, medial orbital | 0 | 0 | 0 | 0 | 2 |
|  | Right Gyrus Rectus | 0 | 1 | 1 | 0 | 1 |
|  | Right Superior frontal gyrus, dorsolateral | 4 | 2 | 2 | 0 | 4 |
|  | Right Middle frontal gyrus | 1 | 1 | 3 | 0 | 0 |
|  | Right Inferior frontal gyrus, opercular part | 0 | 0 | 1 | 0 | 0 |
|  | Right Inferior frontal gyrus, triangular part | 2 | 3 | 3 | 0 | 0 |
|  | Total | 23 | 35 | 34 | 0 | 27 |
|  |  |  |  |  |  |  |
|  |  |  |  |  |  |  |
| Region of Interest | Included Structures | **ADHD SCQ** | **ASD SCQ** | **OCD SCQ** | **ADHD RMET** | **ASD RMET** |
| Other | Left Calcarine fissure and surrounding cortex | 1 | 2 | 2 | 0 | 1 |
|  | Left Cuneus | 2 | 1 | 2 | 0 | 1 |
|  | Left Fusiform gyrus | 1 | 2 | 2 | 0 | 0 |
|  | Left Inferior occipital gyrus | 1 | 1 | 0 | 1 | 1 |
|  | Left Inferior temporal gyrus | 2 | 3 | 5 | 0 | 2 |
|  | Left Insula | 2 | 0 | 2 | 0 | 0 |
|  | Left Lingual gyrus | 0 | 0 | 1 | 0 | 1 |
|  | Left Median cingulate and paracingulate gyri | 1 | 2 | 4 | 0 | 2 |
|  | Left Middle occipital gyrus | 2 | 1 | 3 | 0 | 1 |
|  | Left Middle temporal gyrus | 5 | 5 | 1 | 0 | 0 |
|  | Left Olfactory Cortex | 2 | 1 | 1 | 0 | 0 |
|  | Left Paracentral lobule | 1 | 0 | 1 | 0 | 2 |
|  | Left Parahippocampal gyrus | 1 | 0 | 5 | 0 | 0 |
|  | Left Postcentral gyrus | 1 | 0 | 3 | 0 | 3 |
|  | Left Precentral gyrus | 1 | 3 | 3 | 0 | 3 |
|  | Left Precuneus | 2 | 1 | 6 | 0 | 4 |
|  | Left Rolandic operculum | 0 | 0 | 1 | 0 | 0 |
|  | Left Superior occipital gyrus | 1 | 1 | 1 | 1 | 2 |
|  | Left Superior parietal gyrus | 1 | 1 | 2 | 0 | 3 |
|  | Left Supplementary motor area | 0 | 1 | 1 | 0 | 2 |
|  | Left Supramarginal gyrus | 3 | 1 | 1 | 0 | 0 |
|  | Right Calcarine fissure and surrounding cortex | 1 | 1 | 1 | 0 | 0 |
|  | Right Cuneus | 2 | 2 | 2 | 0 | 1 |
|  | Right Fusiform gyrus | 2 | 0 | 3 | 1 | 1 |
|  | Right Inferior occipital gyrus | 1 | 0 | 2 | 0 | 0 |
|  | Right Inferior temporal gyrus | 2 | 2 | 1 | 0 | 1 |
|  | Right Insula | 0 | 1 | 4 | 0 | 3 |
|  | Right Lingual gyrus | 3 | 2 | 1 | 0 | 0 |
|  | Right Middle occipital gyrus | 2 | 2 | 3 | 0 | 1 |
|  | Right Middle temporal gyrus | 2 | 0 | 1 | 0 | 0 |
|  | Right Olfactory Cortex | 1 | 1 | 3 | 0 | 1 |
|  | Right Paracentral lobule | 2 | 0 | 0 | 0 | 2 |
|  | Right Parahippocampal gyrus | 3 | 2 | 1 | 0 | 0 |
|  | Right Postcentral gyrus | 5 | 2 | 5 | 0 | 4 |
|  | Right Precentral gyrus | 5 | 5 | 2 | 0 | 4 |
|  | Right Precuneus | 1 | 3 | 2 | 0 | 4 |
|  | Right Superior occipital gyrus | 1 | 0 | 1 | 0 | 1 |
|  | Right Superior parietal gyrus | 3 | 1 | 3 | 0 | 3 |
|  | Right Supplementary motor area | 1 | 2 | 2 | 0 | 1 |
|  | Right Supramarginal gyrus | 1 | 1 | 0 | 0 | 4 |
|  |  | 68 | 53 | 84 | 3 | 59 |

ASD: autism spectrum disorder, ADHD: attention-deficit/ hyperactivity disorder, OCD: obsessive-compulsive disorder, RMET: reading the mind in the eyes test incorrect item. SCQ: Social communication questionnaire, social/communication items only. Counts represent number of peak intensities per structure, for each diagnostic group and task. Peak intensities were defined as the statistical maxima within a five vertex radius that remained significant after FDR correction (q=0.05). For precise coordinates and z-values of each peak, data are available upon request. Z-values ranged between -7.7 and 6.7 across groups. **Supplementary Table 7: *OXTR* Allele Frequencies**

|  |  |  | **ASD** |  |  | **ADHD** |  |  |
| --- | --- | --- | --- | --- | --- | --- | --- | --- |
| **SNP** | **Genotype** | **N** | **HWE** | **MAF** | **N** | **HWE** | **MAF** | **1000 genomes Caucasian MAF** |
| **Rs53576** | AA | 11 | Χ^2^= 0.25 | 0.32 | 5 | Χ^2^= 0 | 0.31 | 0.30 |
|  | GA | 41 | p=0.6 |  | 22 | p=0.99 |  |  |
|  | GG | 48 |  |  | 24 |  |  |  |
| **Rs2254298** | AA | 3 | Χ^2^= 5.99 | 0.12 | 1 | Χ^2^= 0.07 | 0.16 | 0.08 |
|  | GA | 16 | p=0.01 |  | 14 | p=0.8 |  |  |
|  | GG | 81 |  |  | 36 |  |  |  |
| **Rs237887** | AA | 38 | Χ^2^= 0.01 | 0.39 | 19 | Χ^2^=0.01 | 0.39 | 0.42 |
|  | AG | 47 | p=0.9 |  | 24 | p=0.9 |  |  |
|  | GG | 15 |  |  | 8 | **χ 2 {\displaystyle \chi ^{2}}** |  |  |

MAF= minor allele frequency, *OXTR*= oxytocin receptor gene, HWE= Harvey Weinberg Equilibrium

**Supplementary Methods for Exploratory Analyses involving *OXTR*:** Genetic data were available on a subset of participants with ASD or ADHD. Three *OXTR* single nucleotide polymorphisms SNPs (rs53576, rs237887, rs2254298) were analyzed on the MassARRAY Analyzer 4 system using iPLEX Gold chemistry (both Agena Biosciences, San Diego, CA), USA using the recommended manufacturer’s protocol; primers and additional methods are detailed in a previous manuscript (34). Ancestry was determined based on caregiver report of the ethnicity of the four grandparents (ADHD: 59% Caucasian, ASD: 63% Caucasian) (34). We examined for associations between genotype and brain structure in brain regions previously found to be affected by *OXTR* genotype in control populations [insula, limbic system, dorsal cingulate, temporal-parietal (lateral) regions] (35-40, 42, 43). We examined both rs53576 and rs237887 using additive models (AA vs. GA vs. GG) but examined rs2254298 using categorical models (AA/GA vs. GG) given the small number of minor allele carriers. We used a linear regression model to estimate the LS mean cortical thickness/ volume measurements as the dependent variable, from sex, age, diagnosis (ASD or ADHD), scanner upgrade, ancestry, and *OXTR* genotype as predictor variables. We tested for genotype-by-diagnosis interactions terms; where non-significant (p>0.05), the interaction was removed from the model and genotype estimates were presented across both diagnostic groups. Where the main effect of genotype was borderline significant (uncorrected p<0.10), we present pairwise genotype comparisons to convey trends.

**Supplementary Table 8: The Association Between *OXTR* genotype and Brain Structure in ASD and ADHD**

| **SNP** | **Region** | **Allele** | **Estimate** | **Lower CL** | **Upper CL** | **F SNP** | **P -value** | **Pairwise** |
| --- | --- | --- | --- | --- | --- | --- | --- | --- |
| Rs53576 | Left lateral group | AA | 4.1364 | 4.0598 | 4.2131 | 3.91 | **0.02** | AA vs. GA p=0.01 |
|  |  | GA | 4.0357 | 3.9959 | 4.0755 |  |  | AA vs. GG p=0.007 |
|  |  | GG^1^ | 4.028 | 3.9867 | 4.0693 |  |  |  |
|  | Right lateral group | AA | 4.1612 | 4.0788 | 4.2437 | 2.55 | **0.08** | AA vs. GA p= 0.03 |
|  |  | GA | 4.0673 | 4.0245 | 4.1101 |  |  |  |
|  |  | GG^1^ | 4.0954 | 4.051 | 4.1398 |  |  |  |
|  | Dorsal Cingulate | AA | 3.66 | 3.57 | 3.74 | 2.17 | 0.1 |  |
|  |  | GA | 3.6266 | 3.5814 | 3.6719 |  |  |  |
|  |  | GG^1^ | 3.5827 | 3.5355 | 3.6299 |  |  |  |
|  | Left Insula ASD^1^ | AA | 4.27 | 4.17 | 4.37 |  |  |  |
|  |  | GA | 4.21 | 4.16 | 4.26 |  |  |  |
|  |  | GG^1^ | 4.18 | 4.13 | 4.23 |  |  |  |
|  | Left Insula ADHD^1^ | AA | 4.45 | 4.29 | 4.63 |  |  | AA vs. GA p=0.0005 |
|  |  | GA | 4.13 | 4.06 | 4.20 |  |  | AA vs. GG p=0.0008 |
|  |  | GG^1^ | 4.14 | 4.07 | 4.21 |  |  |  |
|  | Right Insula | AA | 4.3435 | 4.2422 | 4.4449 | 2.7 | **0.07** | AA vs. GA p=0.02 |
|  |  | GA | 4.2223 | 4.1698 | 4.2749 |  |  | AA vs. GG p=0.05 |
|  |  | GG^1^ | 4.2401 | 4.1855 | 4.2947 |  |  |  |
|  | Left Amygdala | AA | 1196.64 | 1140.33 | 1252.95 | 0.31 | 0.7 |  |
|  |  | GA | 1209.59 | 1179.88 | 1239.29 |  |  |  |
|  |  | GG^1^ | 1196.85 | 1166.39 | 1227.32 | 1.85 | 0.2 |  |
|  | Right Amygdala | AA | 1165.93 | 1113.44 | 1218.42 |  |  |  |
|  |  | GA | 1218.25 | 1190.85 | 1245.64 |  |  |  |
|  |  | GG^1^ | 1207.48 | 1179.65 | 1235.31 |  |  |  |
|  | Hippocampus | AA | 1212.78 | 1146.6 | 1278.97 | 0.78 | 0.5 |  |
|  |  | GA | 1179.08 | 1144.54 | 1213.61 |  |  |  |
|  |  | GG^1^ | 1169.76 | 1134.67 | 1204.86 |  |  |  |
| Rs237887 | Left lateral group | GG | 4.0378 | 3.9736 | 4.102 | 0 | 0.9 |  |
|  |  | AG | 4.0395 | 3.9993 | 4.0798 |  |  |  |
|  |  | AA^2^ | 4.0394 | 3.9944 | 4.0845 |  |  |  |
|  | Right lateral group | GG | 4.0959 | 4.0276 | 4.1642 | 0.10 | 0.9 |  |
|  |  | AG | 4.0814 | 4.0386 | 4.1242 |  |  |  |
|  |  | AA^2^ | 4.0893 | 4.0413 | 4.1372 |  |  |  |
|  | Dorsal Cingulate | GG | 3.6199 | 3.5498 | 3.6901 | 0.19 | 0.8 |  |
|  |  | AG | 3.6139 | 3.5684 | 3.6595 |  |  |  |
|  |  | AA^2^ | 3.6001 | 3.5493 | 3.651 |  |  |  |
|  | Left Insula | GG | 4.1955 | 4.122 | 4.2689 | 0.27 | 0.8 |  |
|  |  | AG | 4.1809 | 4.1348 | 4.2269 |  |  |  |
|  |  | AA^2^ | 4.1677 | 4.1161 | 4.2192 |  |  |  |
|  | Right Insula | GG | 4.3305 | 4.2488 | 4.4122 | 4.06 | **0.02** | GG vs. AG p=0.005 |
|  |  | AG | 4.2094 | 4.1582 | 4.2607 |  |  |  |
|  |  | AA^2^ | 4.2474 | 4.1901 | 4.3048 |  |  |  |
|  | Left Amygdala | GG | 1230.99 | 1187.25 | 1274.72 | 1.81 | 0.2 |  |
|  |  | AG | 1191.59 | 1163.06 | 1220.13 |  |  |  |
|  |  | AA^2^ | 1186.56 | 1155.26 | 1217.86 |  |  |  |
|  | Right Amygdala | GG | 1233.96 | 1192.1 | 1275.83 | 1.02 | 0.4 |  |
|  |  | AG | 1201.94 | 1174.62 | 1229.25 |  |  |  |
|  |  | AA^2^ | 1208.41 | 1178.45 | 1238.38 |  |  |  |
|  | Hippocampus | GG | 1174.7 | 1122.29 | 1227.11 | 0.94 | 0.4 |  |
|  |  | AG | 1165.92 | 1131.72 | 1200.11 |  |  |  |
|  |  | AA^2^ | 1194.36 | 1156.85 | 1231.87 |  |  |  |
| Rs2254298 | Left lateral group | GG^3^ | 4.043 | 4.0072 | 4.0788 | 0.34 | 0.6 |  |
|  |  | A | 4.0275 | 3.9754 | 4.0796 |  |  |  |
|  | Right lateral group | GG^3^ | 4.091 | 4.0529 | 4.1291 | 0.52 | 0.5 |  |
|  |  | A | 4.0706 | 4.0151 | 4.126 |  |  |  |
|  | Dorsal Cingulate | GG^3^ | 3.6177 | 3.5772 | 3.6582 | 1.17 | 0.3 |  |
|  |  | A | 3.5863 | 3.5285 | 3.6441 |  |  |  |
|  | Left Insula | GG^3^ | 4.1752 | 4.1342 | 4.2163 | 0.13 | 0.7 |  |
|  |  | A | 4.1864 | 4.1266 | 4.2462 |  |  |  |
|  | Right Insula | GG^3^ | 4.2441 | 4.1972 | 4.291 | 0.45 | 0.5 |  |
|  |  | A | 4.2207 | 4.1524 | 4.289 |  |  |  |
|  | Left Amygdala | GG^3^ | 1185.52 | 1161.07 | 1209.97 | 6.64 | **0.01** |  |
|  |  | A | 1231.36 | 1195.38 | 1267.34 |  |  |  |
|  | Right Amygdala | GG^3^ | 1203.38 | 1179.75 | 1227 | 2.22 | 0.1 |  |
|  |  | A | 1229.37 | 1194.6 | 1264.14 |  |  |  |
|  | Hippocampus | GG^3^ | 1183.33 | 1153.68 | 1212.98 | 1.35 | 0.2 |  |
|  |  | A | 1157.92 | 1114.28 | 1201.56 |  |  |  |

Estimates are the least squares means thickness (mm) or volume (mm^3^) per genotype group, adjusted for age, sex, diagnosis and ancestry. CL: confidence limit. For volumetric structures, means have also been adjusted for whole brain volume. Children with ASD and ADHD were analyzed together in the same analysis. ^1^For the left insula with rs53576, there was a significant SNP-by-diagnosis interaction (F= 3.14, p=0.05), therefore results are presented separately for the diagnostic groups while adjusting for this effect. For all other SNPs and regions, the interaction term was non-significant (p>0.05) so it was dropped from the model.

References

1. Sled JG, Zijdenbos AP, Evans AC. A nonparametric method for automatic correction of intensity nonuniformity in MRI data. IEEE transactions on medical imaging. 1998;17(1):87-97.

2. Grabner G, Janke AL, Budge MM, Smith D, Pruessner J, Collins DL. Symmetric atlasing and model based segmentation: an application to the hippocampus in older adults. Medical image computing and computer-assisted intervention : MICCAI International Conference on Medical Image Computing and Computer-Assisted Intervention. 2006;9(Pt 2):58-66.

3. Collins DL, Neelin P, Peters TM, Evans AC. Automatic 3D Inter-Subject Registration of MR Volumetric Data in Standardized Talairach Space. Journal of Computer Assisted Tomography. 1994;18(2):192-205.

4. Smith SM. Fast robust automated brain extraction. Human brain mapping. 2002;17(3):143-55.

5. Tohka J, Zijdenbos A, Evans A. Fast and robust parameter estimation for statistical partial volume models in brain MRI. NeuroImage. 2004;23(1):84-97.

6. Zijdenbos A, Forghani R, Evans A. Automatic Quantification of MS Lesions in 3D MRI Brain Data Sets: Validation of INSECT. In: Wells WM, Colchester A, Delp S, editors. Medical Image Computing and Computer-Assisted Interventation (MICCAI98). Cambridge, MA,Verlag Berlin Heidelberg: Springer; 1998. p. 439-48.

7. Kim JS, Singh V, Lee JK, Lerch J, Ad-Dab'bagh Y, MacDonald D, et al. Automated 3-D extraction and evaluation of the inner and outer cortical surfaces using a Laplacian map and partial volume effect classification. NeuroImage. 2005;27(1):210-21.

8. MacDonald D, Kabani N, Avis D, Evans AC. Automated 3-D extraction of inner and outer surfaces of cerebral cortex from MRI. NeuroImage. 2000;12(3):340-56.

9. Chung MK, Taylor J. Diffusion Smoothing on Brain Surface via Finite Element Method. IEEE International Symposium on Biomedical Imaging: Macro to Nano2004. p. 432-5.

10. Robbins SM. Anatomical Standardization of the Human Brain in Euclidean 3-Space and on the Cortical 2-Manifold. Montreal: McGill University; 2004.

11. Lyttelton O, Boucher M, Robbins S, Evans A. An unbiased iterative group registration template for cortical surface analysis. NeuroImage. 2007;34(4):1535-44.

12. Boucher M, Whitesides S, Evans A. Depth potential function for folding pattern representation, registration and analysis. Medical image analysis. 2009;13(2):203-14.

13. Lerch JP, Evans AC. Cortical thickness analysis examined through power analysis and a population simulation. NeuroImage. 2005;24(1):163-73.

14. Ad-Dab'bagh Yea, editor Native space cortical thickness measurement and the absence of correlation to cerebral volume. NeuroImage; 2005; Toronto.

15. Stanley DA, Adolphs R. Toward a neural basis for social behavior. Neuron. 2013;80(3):816-26.

16. Kennedy DP, Adolphs R. The social brain in psychiatric and neurological disorders. Trends Cogn Sci. 2012;16(11):559-72.

17. Reeck C, Ames DR, Ochsner KN. The Social Regulation of Emotion: An Integrative, Cross-Disciplinary Model. Trends Cogn Sci. 2016;20(1):47-63.

18. Alcala-Lopez D, Smallwood J, Jefferies E, Van Overwalle F, Vogeley K, Mars RB, et al. Computing the Social Brain Connectome Across Systems and States. Cereb Cortex. 2017:1-26.

19. Hillis AE. Inability to empathize: brain lesions that disrupt sharing and understanding another's emotions. Brain : a journal of neurology. 2014;137(Pt 4):981-97.

20. Van Overwalle F. Social cognition and the brain: a meta-analysis. Human brain mapping. 2009;30(3):829-58.

21. Baribeau DA, Dupuis A, Paton TA, Scherer SW, Schachar RJ, Arnold PD, et al. Oxytocin Receptor Polymorphisms are Differentially Associated with Social Abilities across Neurodevelopmental Disorders. Sci Rep. 2017;7(1):11618.
